# Local contextual factors of child stunting found via shared values of stakeholder groups: an exploratory case study in Kaffrine, Senegal

**DOI:** 10.1017/S1368980023001088

**Published:** 2023-11

**Authors:** Juan Manuel Moreno, Annabel J Chapman, Chike C Ebido, Ndèye Marième Sougou, Amadou H Diallo, Rahel Neh Tening, Fatou Binetou Dial, Jessica Massonnié, Mahsa Firoozmand, Cheikh El Hadji Abdoulaye Niang, Claire Heffernan, Marie K Harder

**Affiliations:** 1 Values and Sustainability Research Group, School of Architecture, Technology and Engineering, University of Brighton, Brighton BN2 4GJ, UK; 2 Department of Environmental Science and Engineering, Fudan University, Shanghai, People’s Republic of China; 3 Department of Zoology and Environmental Biology, University of Nigeria, Nsukka, Nigeria; 4 Preventive Medicine and Public Health, Université Cheikh Anta Diop (UCAD), Dakar, Senegal; 5 International Research Laboratory (IRL 3189), Environnement santé et sociétés/CNRS /UCAD, Dakar, Senegal; 6 Laboratory of Cultural Anthropology, IFAN, Université Cheikh Anta Diop (UCAD), Dakar, Senegal; 7 Department of Psychology and Human Development, University College London, Institute of Education, London, UK; 8 Faculty of Humanities and Social Sciences, School of Education, Languages and Linguistics, University of Portsmouth, Portsmouth, UK; 9 London International Development Centre, London, UK

**Keywords:** Child stunting, Contextual factors, Shared values, Senegal, Undernutrition, WeValue_InSitu

## Abstract

**Objective:**

This work aims to demonstrate an original approach to identify links between locally situated shared values and contextual factors of stunting. Stunting results from multi-factorial and multi-sectoral determinants, but interventions typically neglect locally situated lived experiences, which contributes to problematic designs that are not meaningful for those concerned and/or relatively ineffective.

**Design:**

This case study investigates relevant contextual factors in two steps: by *first* facilitating local stakeholder groups (*n* 11) to crystallise their shared-values-in-action using a specialised method from sustainability studies (WeValue_InSitu (WVIS)). *Secondly*, participants (*n* 44) have focus group discussions (FGD) about everyday practices around child feeding/food systems, education and/or family life. Because the first step strongly grounds participants in local shared values, the FGD can reveal deep links between contextual factors and potential influences on stunting.

**Setting:**

Kaffrine, Senegal, an ‘Action Against Stunting Hub’ site. December 2020.

**Participants:**

Eleven stakeholder groups of mothers, fathers, grandmothers, pre-school teachers, community health workers, farmers, market traders and public administrators.

**Results:**

Local contextual factors of stunting were identified, including traditional beliefs concerning eating and growing practices; fathers as decision-makers; health worker trust; financial non-autonomy for women; insufficient water for preferred crops; merchants’ non-access to quality produce; religious teachings and social structures affecting children’s food environment.

**Conclusions:**

Local contextual factors were identified. Pre-knowledge of these could significantly improve effectiveness of intervention designs locally, with possible applicability at other sites. The WVIS approach proved efficient and useful for making tangible contextual factors and their potential links to stunting, via a lens of local shared values, showing general promise for intervention research.

Stunting, or low height-for-age, is the most prevalent form of child undernutrition, affecting approximately 149 million children. It impedes them from reaching both their physical and cognitive potential, with known negative impacts to future educational and professional opportunities^(1)^. Pathologies related to stunting and loss of physical growth potential include increased morbidity and mortality, reduced neurodevelopmental and cognitive function and elevated risk of chronic diseases in adulthood^(2)^. Over the past decade, the persistence and increasing severity of global child stunting have driven research to focus on the global objective of reducing the number of stunted children (under 5 years) by 40 % by 2025.

Research has so far focused on four main areas. The first focuses on examining combinations of *pre-defined determinants*, including poor socio-economic conditions, household food insecurity^(3,4)^, poor maternal health and nutrition^(5)^, constraints on women’s decision-making, educational background, antenatal care services^(5,6)^, poor water, sanitation, hygiene, insufficient or late breast-feeding, inappropriate complementary feeding practices^(7)^ and cultural beliefs and socio-cultural realities^(4,8)^.

A second research area focuses on establishing causal links with *specific negative outcomes* of child stunting and linear growth retardation across five domains^(9)^: delayed child cognitive development^(10)^; reduced physical strength and work capacity^(11)^; physiological changes leading to increased risks of adult chronic, non-communicable diseases and mortality^(12)^; increased risk of cephalopelvic disproportions leading to dystocia, mortality and morbidity^(13)^ and undesirable birth outcomes (i.e. low birth weight, small-for-gestational-age infants)^(14)^. Of these, however, causal links have only been established for the latter two domains^(10)^. This focus on causal links is strongly rooted in nutrition research but is reported as inadequate to produce programmes reducing stunting and ensuring children’s full developmental potential^(9)^.

A more recent third line of research focuses on integration of ‘preventive’, ‘support-led’ and ‘growth-mediated’ strategies based on multi-sectoral and multi-factorial approaches^(2,9,15)^. This research explores *how different community resources, capacities and strategies* are fundamental to tackle child stunting, including: social care and health workers’ training and skills^(16)^, mid-level system actors leadership and networking capacities^(17)^, individual and community knowledge, participation and commitment, institutional and organisational capacity to create and maintain partnerships with different stakeholders^(18)^, integration of resource and capital investments on childcare investments embedded in food, education, water, sanitation and hygiene, local governance and business systems^(19)^ and strategic advocacy communication^(20)^.

Across this considerable number of research efforts is the premise that the most effective way to make impact on the consequences of stunting is to intervene to directly improve stunting and linear growth retardation^(10)^. The problem with this direct causal link approach is that it neglects pathways via non-linear, socio-cultural, socio-economic and structural processes and circumstances that contribute to stunting, thus hindering the design of predictably effective interventions.

To improve knowledge on these, recent research has turned to qualitative and mixed-method studies to *explore several indirect influences* of nutrition-related determinants such as gender^(21,22)^, socio-cultural practices and religion^(9,23,24)^, socio-economic^(25)^, structural and environmental factors such as school systems, health communication campaigns, food chains^(24)^, as well as local lived experiences shaping the design, uptake, implementation and efficacy of interventions^(26)^. The social context and cultural meanings within a community shape the perceptions of what is considered a child’s healthy growth^(27,28)^ and influence the engagement in nutrition interventions, such as family relations and gendered household roles^(24,26)^, for example, where women have little control over family finances^(29)^.

We posit that instead of identifying and trying to model an increasing number of candidate factors and then linking them, a more ethnographic approach could be studying local shared values, which permeate local life. We define shared values as those things that people thing are ‘valuable, worthwhile and meaningful’ to groups. Upon these are constructed local perceptions and experiences which carry rich socio-cultural information. For example, in certain settings, moderate undernutrition is perceived not as a health problem but rather a ‘seasonal weight loss’^(24,30)^; the impact of time- and socio-economic costs of participating in interventions reduces their effectiveness^(31)^ and lack of understanding of locally situated perceptions^(32)^ can cause ‘the creation of solutions that are neither meaningful nor beneficial to those in need’^(33)^ ^(pp. 509)^.

We propose that much richer and interlinked contextual detail could be obtained if we started from an understanding of local shared values, and in particular those of groups involved in the socio-economic contexts influencing stunting such as food growing, sourcing, purchasing, preparing and eating, and child education. We therefore adopt the WeValue_InSitu (WVIS) approach for crystallising shared values of groups, immediately followed by a specialised form of focus group discussions (FGD), called ‘Perspectives EXploration’ (PEX:FGD)^(29,34)^, where the information which emerges is grounded in their own mini-culture, and the linking local logic is more apparent. Together, this WVIS_plus_PEX:FGD approach can provide rich information on cultural contextual factors which might influence stunting. Such information could contribute to more culturally acceptable stunting programme design and implementation practices, and localised, meaningful communication strategies^(27,28,31,35)^.

Because this is the first time the WVIS approach is used in health research, we provide some background here on why it was chosen. WVIS provides ‘scaffolding’ to assist local people to better articulate what is ‘important’ to them, in their own way and to the extent of producing statements linked into a framework that can be comprehended by outsiders. WVIS has thus contributed to research and practice in sustainability via local indicators^(33)^, participatory design and strategic planning^(32)^, sustainable development goals^(36)^, action research for building evaluation capacity^(37)^, identifying and assessing legacies of community projects or collaborations^(38)^ and collective meaning-making in community-based development contexts^(39)^. WVIS is a scaffolding process which ensures high face validity^(37)^, which means that the facilitator shows participants how to challenge and clarify their own statements and meaning rather than inserting content for uptake. For those interested in micro-processes, WVIS has been described as complex facilitations to take the group through a tacit-to-explicit translation in cycles of Polanyi-like meaning-making^(40)^.

In brief, this work aims to identify links between locally situated shared values and potential contextual factors of stunting, using an original two-step approach which combines methods of crystallisation of in-situ shared values with specialised FGD which then reveal links to those shared values.

## Method

In this paper, we carry out a case study^(41)^, which utilises the qualitative method WVIS^(29,32–34,36–40)^ to understand local shared values.

Fieldwork and data collection were conducted in Kaffrine, Senegal, in December 2020, in the context of a much larger 5-year project on child stunting: the UKRI GCRF Action Against Stunting Hub (AASH) project. AASH aims to investigate the interrelationship of direct and indirect factors of stunting within and across three communities in which stunting is highly prevalent but for different cumulative reasons: East Lombok, Indonesia; Kaffrine, Senegal and Hyderabad, India (https://actionagainststunting.org/).

The primary unit of analysis was a local group, but with the research design that the data across all groups would be used for a secondary unit of analysis: the shared values of relevant stakeholders in the area of the cohort. Thus, if the participants in one group were too fatigued to be asked the full range of topics, then our data collection ensured that other groups covered them.

**Fig. 1:**
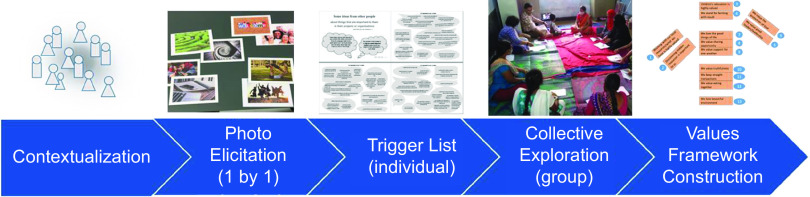
Schematic overview of the macro-level activities carried out during the WVIS workshop session

WVIS resembles a workshop format with set stages (Fig. 1 and Table 1). Preparation is not minimal: it requires pre-localisation of the materials at each site, involving preparation of bespoke ‘trigger lists’ from 4 to 8 local interviews of 20 min each, and approximately 60 h of analysis involving a local researcher. The photo materials are also localised. WVIS must be conducted in the local language and thus requires close collaboration between experienced facilitators and local researchers.

**Table 1:** The stages of the WVIS_plus_PEX approach

Stage		Description
1.1 Pre-WVIS: Creation of localised trigger list		The trigger list is a list of values statements that act as prompts for participants. The list is created through interviewing local researchers and wider groups relevant to the study, and analysis to identify concepts of locally shared values. This local list is then cross-referenced with a globalised reference version to ensure no important values concepts are missing.
1.2 Pre-WVIS: Creation of localised materials		To make the WVIS method more locally appropriate, the usual colour photos were replaced with black and white icons, which were numbered for reference
WVIS main session (2–3 h)	2.1 Contextualisation	During this stage, the boundaries of the group are set, for example, as ‘Mothers in Kaffrine’. This is essential for the following stages: the group must relate their conversations back to this context
	2.2 Localised photo sets	During this stage, participants select from a collection of localised photos to use as a ‘prop’ to communicate what they find, individually, to be ‘worthwhile, valuable and meaningful’ to them about their work in the group. They each present in turn
	2.3 Localised trigger list: selection, discussion and negotiation	During this stage, participants are encouraged to select statements, from the localised trigger list, that resonate with them. This can be done through circling written statements in the list, or statements may be read aloud by facilitators and numbers circled on a bingo card. Participants collectively discuss and develop their joint bespoke statements of what is valued by them: each begin with the phrase ‘It is important to us that…’
	2.4 Creation of Framework and Narrative	During this stage, participants arrange their agreed values statements into a bespoke framework pattern on the table, reflecting their linkages. They then provide a narrative to explain the essence of the framework
Post-WVIS PEX Perspectives EXploration (In-depth Focus Group Discussion session)		This is the optional bolt-on to the WVIS main session, used here to probe for stunting contextual factors. It involves asking the participants focus group interview-style questions; open at first and then more specific. Topics in this case were chosen related to the Hub themes, for example, early childhood learning environment, nutritional habits and perceptions of stunting, as relevant to each participant group, separately

The participant groups were strategically recruited as naturally existing local groups of people whose members have some history of shared experiences (a requirement of the WVIS approach); who live near the main cohort site of the AASH (so that they have relevant shared values) and with some link to the care environment of young children or their food supply (so their FGD would be relevant to the research topic of stunting). Recruitment was conducted by AASH Senegal researchers, resulting in eleven stakeholder groups including mothers, fathers, pre-school teachers, community health workers (CHW), farmers, market traders, farmers and public administration officials (PAO) (see Table 2). All the participants were indigenous to the area. There is no optimal group size other than more than one person and not so many that there is not enough time for every individual to share, that is, 2–18 persons. However, more time is needed for more participants, for example, 2–4 h. To keep the time requested for the participants low, the group sizes were kept small (typically 4 but maximum 12: see Table 2). The questions in the FGD were tailored to the relevance to stunting of each group type: samples are given in Table 2. Ideally, at least two groups of each type would have been recruited so that an indication of variability of results between them could be examined. Recruitment could have been continued until saturation of thematic results was achieved for each group type. However, this work took place during COVID, and local researchers indicated we should proceed with a minimum number of groups. A previous study using the WVIS approach found 6–8 groups each with 2–5 participants sufficient for theoretical saturation of a specific topic^(29)^.

**Table 2:** Participant group types and sample questions for the WVIS_plus_PEX focus group discussions in Senegal

		No. of groups	No. of participants
Mothers	How do you get to market? How do you choose your vendors? Do you buy daily? Store it? What foods should a pregnant woman eat/not eat? Babies? What does a healthy baby look like? Where are the children when you go to market? Who else cares for them? What do children play with? What things should children learn before they grow up? What kind of schools are available?	3	12
Fathers	What kind of food do we use often here? Have your eating habits changed in recent years? What things should children learn before they grow up? What kind of schools are available? From what age? What activities does the father does with the child Would they want to be assisted with eggs to add to their meals	1	4
Grandmothers	(Similar to Mothers) What are good foods for children? What do they cultivate there? Who takes care of the children? What prevents children from growing or having normal height?	1	4
Pre-school teachers	Do you have a classroom or teach outside? What materials do you have? How many children in each group? Do you teach all subjects together or specialised? What do they use to learn how to write? How do you handle the noise? Do you use story telling or role play for any purposes? Are some children difficult to manage? How do you identify stunted children, and do you teach them differently?	1	4
CHW	(Similar to Mothers)	2	8
Farmers	What crops do you grow? Would you wish to grow other things? Do you grow any crops that you didn’t used to grow? Do you employ anyone? Do you grow crops that involve the children? Where do you sell your produce? Do you have problems with insects? Do you store your produce? What is important in the education/training of your children? What do they need to succeed? What kind of teacher would you like to chose?	1	4
Market traders	Do you trade in markets, or stalls or shops? What about mobile selling, using bicycles or motorbikes? What kind of products do you sell? Where do you often get it from? Is the produce nutritious? Tell us about the women merchants	1	4
PAO	How to recognise a child who is malnourished? What are the impacts of malnourishment here? Are there govt programmes for malnourishment? What are the factors influencing malnutrition locally? What is the local perception of malnourished children? Are there any changing trends in food available, or food eaten?	1	4
Total		11	44

The eleven WVIS workshops and PEX:FGD were facilitated by local Senegal AASH researchers, directed by University of Brighton AASH researchers (one on the ground and others online, live), in the local language of Wolof. One of the local researchers was indigenous to Kaffrine: the others were indigenous to elsewhere in Senegal. A live translation into French was audio-recorded alongside the Wolof and later translated into English for initial analysis, along with researchers’ observation notes. Informed consent was obtained from all participants.

### Data analysis

When WVIS_plus_PEX:FGD is typically used to elicit cultural contextual information^(29,34)^, only the summative data outputted by the WVIS are used alongside the PEX:FGD data, that is, the framework of statements of shared values which the group produces as an output (last part of Fig. 1), because they encompass the discussion material. However, in this case, it was found that those statements were abnormally brief, and it was decided to refer to their underpinning discussions within the WVIS where rich, thick descriptions had been made about the group’s cultural context. Thus, transcriptions of both WVIS and PEX:FGD full discussions from the eleven groups were analysed.

The data analysis involved three main steps in a grounded approach^(42)^. First, the transcripts of each group’s WVIS workshop and their PEX:FGD discussions were thematically open-coded for emerging themes, and those themes (with their associated extracts alongside) from all eleven groups were then clustered. Two researchers did this independently in English and compared findings (a local researcher checked findings for integrity of meaning by back-checking with the pre-English versions).

### Findings

**Fig. 2:**
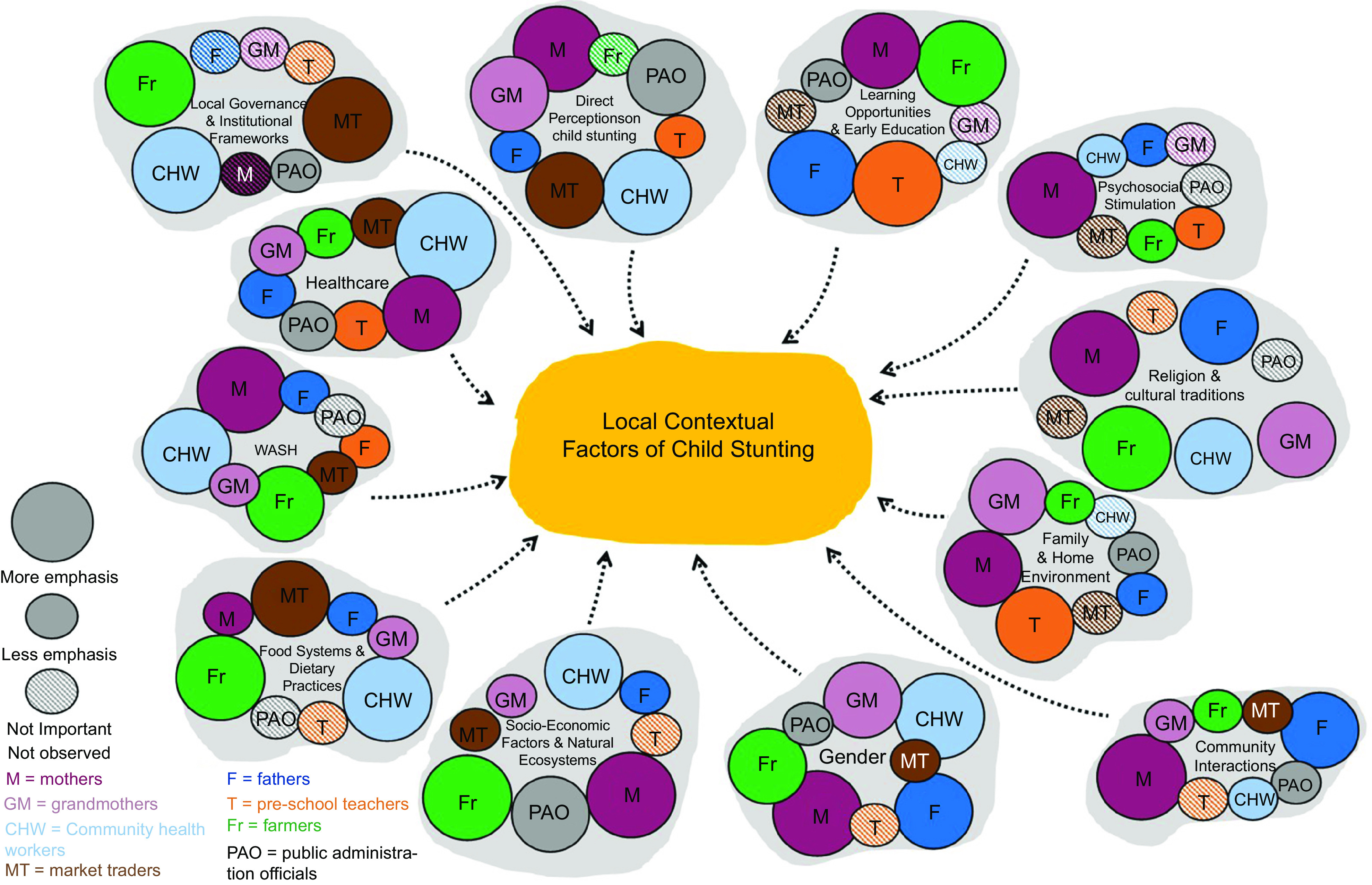
Participant stakeholders’ perceived factors of importance in relation to Child Stunting and Child Overall Development

Across all the participant groups, twelve themes of contextual factors which might influence stunting emerged, presented below. Table 3 provides illustrative quotes. The relative relevance found to different stakeholder group types is indicated schematically in Figure 2.

**Table 3:** Illustrative quotes from participant groups with local contextual factors of child stunting

Local contextual factors of child stunting	Illustrative quotes
Religion and cultural traditions	*[Facilitator/Translator]: When a child is born what do you give him first? [Mother]: Blessed water with Quran writing which are intended to bring him success and blessings[.] Goat milk to make him intelligent[.] Honey.’ (mother, FG #6, Kaffrine, 13·12·2020)* *‘With regard to […] pre-natal consultations there are many factors, including social and cultural, or simply superstitious reasons or shame, or stereotypes, which lead many women to not want to show they are pregnant [… and …] delay their consultation, [even when] godmothers in the neighbourhood try to sensitize and convince them to go get check and profit from pre-natal care.’ (community health worker, FG #3, Kaffrine, 9·12·2020)* *‘[Facilitator/Translator]: Are there other crops you wish you could grow here? [Farmer]: We would like to grow cotton here but unfortunately our soils are not favorable enough for this crop[.] So in addition to cotton[,] we would like to be able to cultivate tobacco but […] religion oppose it[.]’ (farmer, FG#4, Kaffrine, 10·12·2020)*
Gender (roles, responsibilities and expectations)	*‘[…] the lack of means there are some social services in the hospital but it is social service does not work like [.] it is rare to see this service there to help patients and women who cannot afford the least[.] But at the hospital you have to pay to need even when you have a child who is stillborn, even to lift the body, you have to pay first to settle your bill so the social service is really one day really not his role.’ (mothers, FG #6, Kaffrine, 13·12·2020)* *[F/T]: who decides to go to the hospital? [Farmer]: it is the man who must decide; but if the man is not at home, the woman can go to the hospital if the means are there[.] [F/T]: what should be the role of grandmothers? [Farmer]: if the woman is old, her sons have to take care of her[.] It’s not easy to live with a widow, it’s easier to manage than a divorce than a widow.’ (farmer, FG #4, Kaffrine, 10·12·2020)* *‘For the men after they finish selling they can go home and rest whereas the women […] unlike the men who once they have finished their trade in the market can rest and the women do not have this possibility because once they return home they have to ‘other domestic chores that await them and in parallel [must care for] the children to contribute to the functioning of the day.’ (market trader, female, FG #4, Kaffrine, 10·12·2020)*
Healthcare (access to and quality of pregnancy, paediatric and childcare services)	*‘[Facilitator/Translator]: What are the main health concerns of the pregnant women in Kaffrine? [Mothers]: Hypertension, head ache, STI’s, anaemia[.] Women really have problems of lack of finances. [There] are social services in the hospital; but those services rarely attend to women without finances. Even when a child dies at birth they will require money to do the necessary procedure.’ (mothers, FG #6, Kaffrine, 13·12·2020)* *‘The state is always […] the establishment which deals with malnutrition which is called the nutrition service uh it is always at its service to be informed and these services there [for families] that go learn the necessary [on] how children are fed.’ (teachers, FG #3, Kaffrine, 9·12·2020)* *‘The women […] as health worker in the quarters [neighbourhoods] they are often judge[d] by the populations sometimes negative judgment but they really not have to take that into consideration and focus on their job because of they[.] Sometime a man can come an accuse them when their wives maybe may have difficulties in conceiving they men may accuse them of influencing their wives from not conceiving. They always think that the health workers have something to do and influence their wives[.] Sometimes too someone may have a negative believe with regard to their work because people look at them as external figures who come in and influence the women against their traditional believes. So endurance is a very vital quality for them to have as health care workers.’ (CHW, WVIS #3, Kaffrine, 9·12·2020)*
Food systems and dietary practices	*[Facilitator/Translator]: When should diversification in child food start? [Grandmother]: As from 3 months foods like cassava and other cereals[.] [F/T]: Does the children eat with parents or not? [Grandmother]: From one year the child eats with the parent but in case the child has bad table manners they will serve him food aside but otherwise they all eat together. [F/T]: What are good food for children? [Grandmother] potatoes, carrots, banana, eggs, eggplant, potato, fish, meat, vegetables[.] They don’t eat eggs before the child starts speaking (the child only eats eggs when he starts talking)[.] This is because its very heavy and can cause bloating and may also lead to intestinal problems[.] [F/T]: What other foods should not systematically give to children? [Grandmother]: Millet, fufu(couscous).’ (grandmother, FG #2, Kaffrine, 8·12·2020)* *‘Some aliments are forbidden for pregnant women (watermelon, would increase the amount of amniotic fluid), sour aubergine, grilled red meat (would cause bleeding), a specific type of sea food (would cause the child to dribble) and too much eggs (would cause overweight in children).’ (community health worker, FG #1, Kaffrine, 7·12·2020)* *‘If [we] had the means and the buying power is also very low so they are forced to sell fish of low quality […] when the fish of good quality arrives, it is taken to big cities like Dakar where the people have the ability to buy and the low grade fish are sent to small villages [like Kaffrine]. […] [We] sometimes have shortages when the fishermen decide to go on strike. So not only [we] get the bad qualities but also have shortages. [We] can take fish on credit and also sell on credit. ‘ (market traders, WVIS/FG #4, Kaffrine, 10·12·2020)*
WASH (water, sanitation and hygiene)	*‘[Facilitator/Translator]: Where do the families get water from? Are there health problems with some of the water sources? [Mother]: We drink water from the tap we pass it through filter if we have no means to buy. [F/T]: Do the children typically stay around animals? [Mother]: Yes we have goats, ships and rabbits so sometimes the child should be beside the animals but they don’t touch them apart from the rabbits where they can play with their ears. [F/T] Do the children help with jobs like collecting dung? Animal care? [Mother]: Yes[.] [F/T]: Wear shoes? [Mother]: […] sometimes they walk [barefooted] but that’s just indoors.’ (mother, FG #6, Kaffrine, 13·12·2020)* *[Facilitator/Translator]: are you considering other crops? [Fr]: we want to cultivate millet, sap, but the land is not fertile[.] we would like to market gardening but we do not have access to water.’ (farmer, FG #4, Kaffrine, 10·12·2020)* *‘That we have better amenities in the market and canals for evacuation for the better management of waste[.] That is because there are no canal systems to evacuate the fish and other things so [we] will like to have more amenities that are convenient.’ (market traders, WVIS #4, Kaffrine, 10·12·2020)*
Socio-economic factors and natural ecosystems	*‘[S]ome of [us] have [an] appointed seller but others sell buy any seller without having a preferential seller supplier[.] [F/T] why did you choose to have an appointed seller? [Mother]: because [there are] times […] you [need] to benefit from a credit or an advance and therefore it is more practical […] to have to buy the same seller to be able to benefit from this service in case [you] needs a loan by example or advance of goods. [F/T] Another reason why you prefer a trader or a seller? [Mother]: yes indeed we can choose to go to a seller because he is related with us[,] we can also choose the seller because he will go produce quality[,] or else we can choose to go to a seller simply because he is found to be friendly and uh well, well or when he is willing to come to us in case we do not have enough money for example.’ (mother, FG #5, Kaffrine, 11·12·2020)* *‘It’s important to have financial means to meet our needs and be able to get good health and good education for their children[.] Its important to have projects that can enable us to work and and earn money so we can provide good food for our families[.]’ (grandmother, WVIS #2, Kaffrine, 8·12·2020)* *‘[Facilitator/Translator]: Is this agriculture essentially one where partially subsistence agriculture? [Farmer]: Yes, the crops that we get from agriculture partly allow us to supply us with food whether it is millet corn peanuts and other products. [F/T]: have you recently introduced a new sector in your agricultural practice? [Farmer]: Yes, now we are growing more and more chilli peppers and Malian Shepherds whereas before that was not done here so it is quite a novelty that is has become an integral part of our bag of our agricultural activity so we also cultivate okra whereas before that was not done here. You have found the 10 there is a food innovation here that is to say a food that you consume them you have started to consume recently and that did not exist before. [F/T] Harvest here in Kaffrine or do you export it elsewhere? [Farmer]: for our school at the local Kaffrine market here a Kaffrine and we do not export elsewhere. we also sometimes reserve part of our school for the time of smiling or even need money we use it to have money the same goes for the cattle the goats the sheep that we can sell a gift need to have some money. no no let’s not change with the big traders everything we produce we sell it here in Kaffrine.’ (farmer, FG #4, Kaffrine, 10·12·2020)* *‘[…] it is not so much the availability of products […] that poses a problem as [is] the question of the financial means to obtain these products.’ (PAO, FG #5, Kaffrine, 11·12·2020).* *‘In terms of climate change it is obvious that […] is a phenomenon […] that’s not beneficial for us[.] That there [is] the agriculture the season is short or that the later [rains] arrive[,] it will inevitably impact the yields and suddenly influence the food and the diet[.]’ (PAO, FG #5, Kaffrine, 11·12·2020)*
Learning opportunities and early education	*‘[Facilitator/Translator] what would need to be done so that a child can prepare for a living to be successful professionally? [Farmer] […] the ideal for a child is to attend the Franco-Arab school […] because if you send your child to the French public school she will have less chance of acquiring a culture pretty young people of rudiments of religion of Islam it will pay to make its applications accomplish duty of what and if you send it in there ‘Koranic school simply, it will not be able to increase its chances of having a future professional integration so the good compromise would be to send them to schools where Arabic and French are taught at the same time.’ (farmer, FG #4, Kaffrine, 10·12·2020)* *‘[It is important to educate] young girls and keeping them in school […] at this level and has a lot of work to do because early marriage prevents some girls to be able to stay in school and therefore it would be very important to emphasize this aspect uh equal opportunities are respected.’ (PAO, FG #5, Kaffrine, 11·12·2020)*
Family and home environment	*‘[Facilitator/Translator]: Who takes care of the children? [Grandmother]: The grandmothers[.] Even when the mothers are at home they will send the children to the grandmothers to play with and have that affection (families always consist of so many persons)[.]One grandmother can sometimes find her self with so many grandchildren to take care off as a result of polygamy.’ (grandmothers, FG #2, Kaffrine, 8·12·2020)* *‘[…] the family is something that is very close to my heart every time I see a child I’m already trying to think in my head about all his family […] [it] brings me back to the idea of family which is an idea that is very dear to me[.] And another aspect which is characteristic of this image is that we find that the child is well surrounded and that he is safe between his 2 parents […] [This image] represents a moment and we have a mom and a dad who bring their children to school and it shows that the education of children is particularly represented in this image I am a person who thinks that any child has the right to an education and therefore the […] simple fact of seeing children who are supervised and who benefit from this right is something that I find very positive and very encouraging.’ (teacher, WVIS #3, Kaffrine, 9·12·2020)* *‘family[,] the family at the base […] everything in everyone must be in a family setting […] within a family environment then health[,] the food[,] the […] good food[,] good nutrition […] what [is] at the base we find the family environment and food being within a family.’ (PAO, FG #5, Kaffrine, 11·12·2020)*
Psychosocial stimulation	*‘[Facilitator/Translator]: We heard some families with very few children, might take food to families with many children, so they will be stimulated to eat better. Is this common? [Mother]: Yes it happen that we use that strategy so that children can eat. Note that children like to Imitate so that’s why we do that.’(mother, FG#6, Kaffrine, 13·12·2020)* *‘The universe where everything goes through the game […] develops the creativity and the spirit of the children and therefore the ball which is represented on this image there is particularly important for me for to do a good teaching you have to go through games and playfulness.’ (teacher, WVIS #3, Kaffrine, 9·12·2020)* *‘Meals are diversified but children cannot touch what is in the middle of the plate (and it is usually where the most nutritious food is placed). Children have to be served and cannot take the initiative to serve themselves.’ (community health worker, FG #1, Kaffrine, 7·12·2020)*
Community interactions (collaboration, solidarity, support and conflict)	*‘[F/T]: where do you leave your kids if you should go somewhere? [Mother]: At home with our mothers-in-law and neighbors’ (mother, WVIS #5, Kaffrine, 11·12·2020)* *‘[It is important for us] to play an intermediary role between the population and medical practitioners[.] To play a role of follow up to the population. This follow up consist of going to mothers, breastfeeding mothers, young girls to remind them of all the what they should do as pers the recommendations for health officials.’ (CHW, WVIS #3, Kaffrine, 9·12·2020)* *‘[there is] conflict between uh between farmers and uh herders the herders whose animals are often wandering between the fields and […] it is a source of tension between the farmers and the uh breeders they have to be on their guard all the time to be able to watch their fields because the animals are constantly wandering and it often happens that there are scuffles between the peasants and the herders and the latter are often armed […] it is not uncommon to see incidents happen er between the farmers and the herders.’ (farmer, WVIS #4, Kaffrine, 10·12·2020)*
Local governance and institutional frameworks	*‘[we believe] the Government does not help [us] when it comes to nutrition/access to food.’ (community health work, FG #1, Kaffrine, 7·12·2020)* *‘It is no longer possible to [get] a loan. You can use goods to benefit from seeds. Access to equipment is also important. Beyond that, we also want food aid, equipment such as seeders, cart, sunna 2 and 3 for the seed. [We] also crave off-season agriculture. […] The ballot issue [government elections?] also handicaps us. The state is also struggling to get stocks on time.’ (farmers, FG #4, Kaffrine, 10·12·2020)* *‘To be members of an economic to be able to obtain financial assistance[,] To have micro finance credit union amongst women sellers [Translator note: participant explains having benefited from government assistance by taking part in some financial grouping].’ (market traders, WVIS #4, Kaffrine, 10·12·2020)*
A separate category: Direct perceptions on child stunting (specific perceptions of stunting which arose in the data)	*‘[Facilitator/Translator] How do you recognize a healthy child [?] [Mother]: by his weight and height at birth, cries [.] There is a standard scaling […] every month [of] the child for the height and weight to […] measure[.] Apart from height and weight[,] we recognize a child who is not in good health when he cries a lot, his very soft pale face, lack of appetite, stays so connected [dependent] to his mother.’ (mother, FG #6, Kaffrine, 13·12·2020)* *‘When the child has a fever, when he has the [cough] and when he is inactive he plays not.’ (father, FG #2, Kaffrine, 8·12·2020)* *‘[We] cannot assess them [most cases of malnutrition in Kaffrine] because they do not all come to the health structures. They are frequent but they are not notified. You have to see the height and weight to know the health status of the child. You also have to make a correlation to see the child’s illness. The major problem is related to training’ (CHW, WVIS #1, Kaffrine, 7·12·2020)* *‘The consumption of some kind of fish can affect the child’s intelligence and health and are also a source of illness and [we] always sell those kinds of fish which are not nutritive because they are cheap.’ (market traders, WVIS/FG #4, Kaffrine, 10·12·2020)* *‘cause of malnutrition the answer is uh it is poverty that is at the root of malnutrition because apart from parents who do not have enough money or otherwise in the activities do not allow them to have enough resources will have difficulty feeding their families well so it is the situation of poverty that is the first explanatory factor of malnutrition here in Kaffrine.’ (PAO, FG #5, Kaffrine, 11·12·2020)*

### Religion and cultural traditions

For *parents, grandmothers, teachers* and *farmers,* religion and the maintenance of cultural traditions were core to children’s education, to develop a culture and moral life path of respect, solidarity and cooperation within the community.

For *mothers* and *grandmothers*, religion had an important role in nutrition, concerning recommended and prohibited foods, breast-feeding and seeking antenatal care.

*Farmers* mentioned diversifying food crops could ensure more secure livelihoods: ‘*we would like to be able to cultivate tobacco but […] religion oppose it[.]*’ (Table 3). They mentioned praying as a strategy to deal with bad climate and pests.

*CHW* saw the observance of certain religious practices and beliefs as detrimental towards nutrition in pre-natal and post-natal diets. They thought the stigma around close pregnancies and religious and cultural practices also discouraged women from seeking professional help and advice during the early pregnancy; ‘[…] *simply superstitious reasons […]lead many women to not want to show they are pregnant […]*’ (Table 3).

### Gender (roles, responsibilities and expectations)

Gender was mentioned relating to food planning and nutritional strategies, access to and use of healthcare services during pregnancy and for educational and income-generating opportunities.

Responses from *parents*, *grandmothers* and farmers indicated a clear gender-biased division of labour that extended beyond home environments. *Mothers* and *grandmothers* mentioned that they took charge of meal planning, housework and childcare, but their management choices were constrained due to lack of control over household finances; irregular income of their husbands and the lack of opportunities to access jobs.

*Fathers* affirmed that women oversaw housework and food planning, and household management spending. Some *fathers* suggested the work of *mothers* needed to be further recognised; others talked about women’s role in society as ‘reproductive function’ activities *v*. men’s ‘production’ activities.

*PAO* referred to the difficulties for women and especially young girls to attain higher educational levels due to socio-cultural pressures for marriage and early pregnancies.

*CHW* emphasised that women had limited or no control over household finances and their difficulties in accessing jobs seriously affected their autonomy and capacity to access health care. This was compounded by women’s limited autonomy to seek professional advice during the early stages of pregnancy, depending on the consent and predisposition of their husbands and/or extended families.

### Health care

*Fathers* were concerned with access and quality of health care and hospital births. *Mothers* and *grandmothers* mentioned hospital check-ups and following doctors’ advice as requisites for a ‘good pregnancy’: they also said they often could not afford it. *Teachers* emphasised the importance of the health services to carry out regular trimestral checks on children and give nutrition advice.

*PAO* emphasised the importance of hospital births not only for health and hygiene reasons but also for administrative purposes since hospital registration produces certificates necessary for subsequent access to government aid education.

For *CHW*, women’s limited independent decision-making powers and health care access during early stages of pregnancies had important consequences for their family planning decisions, nutritional knowledge and childcare capacity. Inadequate pregnancy care and early childcare practices were further hindered by lack of knowledge and awareness about child stunting, which itself sometimes led to negative attitudes and stereotypes not only towards stunted children but also community doctors and health workers, especially female, who were sometimes blamed for pregnancy complications.

### Food systems and dietary practices

Food systems and dietary practices were relevant to most stakeholders, shaped by several converging factors.

For *mothers*, food planning and nutritional strategies were based on day-to-day decisions like food prices, household budget and storage conditions (e.g. owning a fridge to keep foods fresh) which often superseded nutritional and hygiene considerations. Other influences were religions recommendations for food types and breast-feeding.

*Parents* and *grandmothers* gave conflicting comments on whether eggs were good or bad for pregnant women and/or babies: some believed it could impede a baby’s speech development and some did not. They were open to interventions providing eggs but disagreed on whether the eggs would be eaten daily or stored instead.

*Teachers* mentioned concerns over food affordability and hygiene at schools, saying most parents chose to cook children’s lunches at home.

*Farmers* worried about agriculture becoming a less-secure livelihood. Income diversification was influenced by religious observance (not to cultivate certain crops like tobacco and using prayers for rain and pests) and geographical and climatic constrains (limited fertile soil and lack of water to cultivate watermelons or cotton). *Farmers* said difficulties to access loans for equipment, storage facilities and fertilisers, restricted their farming choices.

*Market traders* referred to the poor quality of fish they could acquire to sell in Kaffrine’s market, which could hinder children’s health and cognitive development, saying that better fish went to bigger/richer cities.

*CHW* mentioned that religious and cultural beliefs around food practices could influence the nutritional quality of household diets strongly, including breast-feeding and food diversification. They echoed *farmers’* and *market traders’* concerns on food supply chain constraints, competitive markets, food storage limitations and climatic and seasonal factors.

### Water, sanitation and hygiene

For *parents* and *grandmothers*, access to water was a main concern for the choice and preparation of foods, and hygiene.

Water scarcity was rated important by *farmers* for cultivation of choice crops like watermelon, cotton and for sanitation for *market traders* to evacuate food waste to drains. *CHW* commented that insufficient access to water impacted on nutritional strategies and health, including stunting.

### Socio-economic factors and natural ecosystems

For *parents* and *grandmothers*, livelihood insecurity, access to nutritious foods at affordable prices in local markets, limited transport and infrastructure to access water, limited or distant food markets and lack of fridges/storage to preserve food were all mentioned.

For *teachers*, work conditions influenced their role in early education of local children, motivational (remuneration), capability (facilities and resources) and capacity (pedagogical and professional training) terms. *PAO* considered poverty as the main cause of child stunting and malnutrition, saying families simply did not have enough income and resources.

*Farmers* stated that agriculture had become an insecure livelihood due to bad climate, market speculation, water scarcity, infertile soils, lack of and difficulty to access government loans and insufficient means to pay a workforce. This meant that their families, including children, worked in the fields to support the household. *Market traders* said that their low purchasing power and bureaucracy prevented them from improving and diversifying their livelihoods.

### Learning opportunities and early education

As stunting is linked to reduced learning, local perceptions on learning are relevant. Most stakeholders indicated early learning opportunities as important for overall child development.

While for *mothers* the emphasis was access to (any) good education, *fathers* specifically considered education for its value for future professional opportunities and livelihood security to permit children to ‘do better than their parents’.

*Farmers* mentioned that children’s contribution to society required an education that was comprehensive and including Quranic/Muslim values for a culture of morality and dignity, and the technical elements of French schools, for better professional opportunities.

*Teachers* mentioned difficulties for some families to afford early education. They emphasised the importance of parental relationships and deep engagement with their children prior to joining formal education for their development, as was ongoing communication between teachers and parents. *Teachers* said a varied syllabus and wide range of educational resources and materials was key to nurturing children’s cognitive and psychosocial development and emphasised the need for better and repeated pedagogical and relevant professional training for their motivation and capabilities as teachers.

*PAO* linked low educational attainment of girls and women to gender and socio-cultural pressures and stated teenage extra-marital pregnancies caused school dropouts.

### Family and home environment

The immediate and extended family environments were emphasised by many groups.

For *mothers*, the focus was making sure that parents and grandparents were actively involved in enhancing child development, getting children interested in learning and participating in daily chores.

The idea of unity within the household – ‘togetherness’ – was stressed by *fathers* and *grandmothers*. *Fathers* mentioned negative impact of divorce, and associated risks of children being neglected. *Grandmothers* described their roles including supporting their children’s families financially and in-kind; caring for grandchildren and sharing their knowledge and experience. They explained how developing ‘relations capital’ (networking) was very important to create opportunities of support for their extended families.

Both *parents* and *grandmothers* considered pre-marital and early-age pregnancies negatively, as they affected families’ dignity, carried financial constraints and increased difficulties to register children at birth – a prerequisite for accessing education and health care.

### Psychosocial stimulation

For *fathers*, learning early how to pray, carry out ablutions and accompanying parents to the Mosque were important for moral and social development, while *mothers* said that having their children learn to read the Quran developed language comprehension and cognitive skills. Playing, storytelling, role-playing and sports were also all considered important for development.

*Teachers* emphasised the importance of religious education for psychosocial stimulation and to help develop harmonious and cooperative community relations. *Teachers* and *mothers* mentioned activities with neighbours, such as cleaning of common places or sharing meals, for children’s social development. *CHW* said sharing meals was sometimes a strategy for slightly better-off families to help others and/or to stimulate children to eat better.

### Community interactions (collaboration, solidarity, support, conflict)

Although considered an indirect impact on child stunting, respect, solidarity and collaboration within the community were mentioned by all stakeholders.

For *parents*, the process of collaboration between members of the community involved a sense of belonging, ‘togetherness’ and ‘peacefulness’, especially when nurtured through collective actions (taking care of other families’ children, sharing meals, cleaning neighbourhood common areas, traditional celebrations).

*Mothers* and *grandmothers* added that it was through community meetings and mothers’ groups that spaces and opportunities for sharing practical knowledge and advice about parenting and childcare were created. For *mothers, farmers* and *market traders*, such spaces created a sense of not only belonging but also mutual support and solidarity. This included sharing meals and helping each other out financially or in-kind when needed. For *farmers* and *market traders*, it was a strategy to strengthen their livelihoods during bad seasons, or when resources were scarce.

*Teachers* emphasised child involvement in community activities, celebrations and collective collaborations, to help them become ‘exemplary citizens’.

For *CHW* and *PAO*, good community relations depended on the development of sustainable cross-sectoral services for better provision of health care, education and local governance, and their role as mediators was to ensure these collaborations happened.

### Local governance and institutional frameworks

Local governance, civic administrative and institutional frameworks were mentioned for important, but more-indirect, impacts.

*PAO* highlighted the importance of registering births in hospitals, to facilitate later formal access to essential public services. *Fathers* and *farmers* said it was a prerequisite for their children to access schools. *Fathers* added that it facilitated mothers’ access to childcare and nutrition training.

For *farmers* and *market traders*, joining formal microcredit unions gave them access to governmental aid, and thus support to strengthen and diversify livelihoods, via storage facilities and loans. For *market traders*, especially women, formalising their trade increased their chances of accessing financial business support.

*Teachers* considered local government responsible for, but not delivering on, improvement and development of much-needed school resources, working conditions and relevant pedagogical and professional training, which they felt influenced their role in maximising children’s development potentials.

*CHW* and *teachers* emphasised the importance of good leadership and efficient local governance through cross-sectoral collaboration and better communication between mid-level actors to draw resources, raise awareness, share knowledge and implement health interventions aimed at reducing infant and maternal mortality rates.

### Perceptions on child stunting

In addition to the above contextual factors which emerged from more open and broad questions, our analysis also drew out some *directly articulated local perceptions of stunting*, which we summarise below.

Among *parents*, and *teachers* and *grandmothers*, child stunting was mostly perceived as a health problem impeding a child’s physical and cognitive development and was primarily recognised through physical features (short stature, low weight, hair loss) and attitudinal and behavioural signs (fatigue, lack of concentration, bad temper, unsocial behaviour, lower school performance). *Teachers* mentioned ‘child health check cards’ provided by the local health departments to measure the children and twice-a-year formal visits from health workers.

For *grandmothers*, stunting was considered due to lack of hygiene; spiritual influences during pregnancy; possibly hereditary and/or food preparation practices such as leaving food uncovered where spirits could enter. *Teachers* considered the family context to be relevant, including broken marriages and parental attitudes towards such as overprotection or dismissal of the issue of the stunting, due to lack of knowledge.

*Market traders* talked about the poor quality of fish sold in their market and the lack of hygiene and water facilities to drain out waste, as potential causes for children’s health problems: they said poor quality fish caused intestinal worms and food poisoning and hampered children’s intelligence.

*PAO* mentioned poverty and lack of income opportunities for families, leading to only affording low food nutritional quality, as main causes of child stunting.

Some *mothers* expressed not fully understanding the concept of stunting. Any existing knowledge and strategies originated from mothers’ meetings and extended family circles. PAO mentioned that, to their knowledge, there was no specific government plan or strategy on child stunting or related nutrition. CHW described a wide range of factors of stunting and undernutrition including poor diets, disease, poor hygiene, hereditary factors, socio-cultural beliefs and practices around food, non-compliance with exclusive breast-feeding and premature pregnancies. Eating of prohibited foods and/or neglecting correct foods were perceived to lead to ‘good’ or ‘bad’ pregnancies, affecting whether the babies would be stunted. CHW also mentioned that it was difficult to diagnose stunting and malnutrition cases as people did not come regularly for check-ups.

## Discussion

Our findings make contributions to two complementary strands of child stunting research: those highlighting the need for *more relevant* determinants and outcomes of stunting^(9)^, and those calling for *more multidisciplinary approaches* that take into consideration not only nutrition-specific determinants but also *cross-sectoral factors* that underlie nutritional challenges^(2)^.

First, while they remain exploratory in nature and limited geographically and culturally to the context of Kaffrine, Senegal, our findings provide direct insights of local culturally contextual factors which could influence child stunting and development, drawn from discussions grounded in the shared values of local community groups with roles relevant to child upbringing, education and care (mothers, fathers, grandmothers and pre-school teachers), as well as health, local governance and the food chain (CHW, farmers, market traders and PAO).

These contextual factors included unawareness of stunting as a problem, lack of information about nutrition (e.g. limited community nutrition training programmes, lack of school resources and specialised pedagogical training of teachers); religious and spiritual beliefs and cultural traditions concerning certain food eating and growing practices; women’s lack of autonomy to access healthcare services during pregnancy, school education dropouts and limited financial and income opportunities; male-dominated authority and decision-making within the household pertaining food budgeting and education; lack of trust and stigma towards health workers for pregnancy; socio-economic factors affecting children’s food environment (e.g. school meals, household irregular income, price of quality nutritious foods, lack of fridges to keep foods fresh, water, sanitation and hygiene conditions in markets and homes) and structural factors affecting the food chain (e.g. availability and quality of foods sold at markets, climate change, access to water and soil fertility to grow desired crops, storage facilities, access to micro-loans to support small merchant and farm businesses). Pre-knowledge of these will allow the design of more locally acceptable and effective interventions in Kaffrine and flag new themes of contextual factors for consideration at other sites.

Secondly, the approach we have used provides links between these candidate factors and the complexity of daily life: contextual links. In the introduction, we outlined a large body of child stunting research, which tends to be persistently framed in terms of short-, medium- and long-term negative outcomes^(9)^, investigating potentially causal links between linear growth retardation and child stunting, and academic sourced factors from discipline-specific knowledge bases. This framing is problematic not only because it is insufficient to ensure overall child development and/or nutrition, but also because it implies that stunting interventions are failures even if they can significantly improve children’s situation^(9)^. Furthermore, this focus on linear growth retardation and stunting has meant that efforts are directed towards the first 2 years of life window, when growth failure is most rapid, and interventions are most likely to have an impact^(9)^, to the extent of neglecting related aspects of nutrition, health and overall child cognitive, physical and psychosocial development which extend well beyond that first 2-year window, into adolescence^(9)^.

Our examination of local perceptions of stunting and its contextual factors is also of relevance to larger existing frameworks, such as WHO’s 2013 conceptual framework Context, Causes and Consequences of Childhood Stunting (‘WHO Stunting Framework’)^(43)^, as it helps identify missing interlinking factors that are directly relevant to local communities and thus could be used to create more specific indicators. For example, while the WHO Stunting Framework includes indicators for ‘Home Environment’ under its ‘Household and Family Factors’ section, it does not provide specific factors that have been reported to affect a child’s birth and/or growth development such as family members relationships, gender norms or household socio-economic characteristics. While some of these missing factors have already been reported^(44)^, our value-based approach provides detailed understanding of locally important contextual factors not previously considered.

As Leroy and Frongillo^(9) (pp. 202)^ point out, a major pragmatic challenge is that most interventions fail to pinpoint and then address factors of child stunting or the type of interventions needed. The integration of multiple types of interventions spanning nutrition and other underlying contextual causes is needed^(9)^. This paper demonstrates that the WVIS approach is effective and useful for crystallising/creating local knowledge, allowing us to identify and articulate specific practices, motivating factors and strategies which are relevant to local community stakeholders in the context of child stunting and undernutrition. It is a highly localisable and culturally adaptable approach^(29,32–34,36–40)^. WVIS produces statements of core shared values of the groups, which provide a weft onto which they can weave a communicable context-rich picture of their perceptions of stunting. This picture is inherently multi-factorial: concepts are framed in local values, transcending disciplinary boundaries. But they can be interrogated subsequently in terms of disciplinary frameworks.

This work thus demonstrates a holistic approach (WVIS_plus_PEX:FGD) to generating grounded, multi-factorial and multi-sectoral determinants of stunting, and reveals their pathways within local contexts. Application at other sites will allow comparisons and patterns of such contextual factors to be built up, contributing to an understanding of factors of stunting in those sites. The AASH project presumes that there is not one cause nor one type of stunting but a typology with different active factors in different contexts, and it is collecting a wide range of objective data – genetic, biome, gut health, diet, nutrition, food environment, food systems, child cognition and water, sanitation and hygiene – across Indonesia, Senegal and India sites, to try develop the specifics of a typology. The WVIS_plus_PEX:FGD shared values data from each site will be used to help understand possible cultural linkages between those objective data, and to inform intervention planning.

In the introduction, we drew out the trends in current research in child stunting of moving from studies of single and direct factors to consider multi-factorial issues. When we designed and wrote this study, we envisaged the shared values of local groups to be mini-cultures which, if probed appropriately, could provide not only more complete and authentic contextual factors but also an underlying understanding of why they existed in that place. We did not have anthropological training but came from a background of developing authentic indicators for local sustainability^(33,36)^, co-developed over years of action research^(37)^. But reviewer feedback pointed us to related anthropological work, and we now realise that the WVIS_plus_PEX:FGD approach has much in common with the established anthropological concept of cultural schema^(45)^, the methods of focused ethnographic studies^(46)^ and community-based participatory research^(47)^ and that these have been applied in nutrition studies already^(27,28,48)^.

Both WVIS and focused ethnographic study retain the characteristic of traditional ethnography in their intent to obtain the emic view within communities^(41,48)^, while aiming to reduce the time taken to attain cultural data compared with traditional ethnography^(46,48)^. However, they do this in diverse ways. Focused ethnographic study focuses the questions on the topic under study^(48)^ such as an intervention or policy design and planning^(49)^. WVIS uses the mechanisms within the highly reflective and introspective workshops sessions which involve tacit-to-explicit translations^(40)^, to facilitate deep discussions about shared experiences and articulate them into statements reflecting what is ‘important’ to that group (emic). Only after this is a FGD used to elicit information on a specific topic. WVIS can thus be used to address a broader range of research questions. Furthermore, the cycles of meaning-making and introspection involved facilitate deep, authentic participation^(34)^, which reduces the risk of tokenistic or ‘tick box’ engagement that is common pitfall in community-based participatory research^(48)^.

Future work will include investigating in detail the overlaps between the approaches. We note that the WVIS_plus_PEX approach includes the notion that each type of stakeholder group has their own mini-culture, which is particularly effective at providing a boundary of contextualisation which can facilitate the articulation of information which is intricately linked to the shared values of that mini-culture. If true, this would be an interesting development for cultural schema, and the short time needed for WVIS_plus_PEX of 2–4 h might prove it a useful accelerated quasi-anthropological method: this can be investigated in future work.
